# Risk of Revision and Patient-Reported Outcomes After ACL Reconstruction: Influence of Concomitant MCL Injury and Graft Choice: Analysis of 35,139 Reconstructions From the Norwegian Knee Ligament Register

**DOI:** 10.1177/03635465261457327

**Published:** 2026-06-29

**Authors:** Thomas Birkenes, Jorge Chahla, Stein Håkon Låstad Lygre, Andy Williams, Eivind Inderhaug

**Affiliations:** † Department of Orthopaedic Surgery, Haukeland University Hospital, Bergen, Norway; ‡ Sports Trauma and Arthroscopy Research group, Bergen, Norway; § Department of Orthopaedic Surgery, Rush University, Chicago, Illinois, USA; ‖ Department of Occupational Medicine, Haukeland University Hospital, Bergen, Norway; ¶ Fortius Clinic, London, UK; # Department of Biomechanics, Imperial College, London, UK; Investigation performed at Haukeland University Hospital, Bergen, Norway

**Keywords:** anterior cruciate ligament, knee ligaments, medial collateral ligament

## Abstract

**Background:**

Concomitant medial collateral ligament (MCL) injury is common in anterior cruciate ligament (ACL) tears, but optimal management of these injury components remains controversial.

**Purpose:**

To compare patient-reported outcome measures and the risk of ACL revision surgery in patients with concomitant MCL injuries (treated operatively and nonoperatively) versus isolated ACL injuries and to assess whether MCL treatment strategy or ACL graft choice influenced outcomes.

**Study Design:**

Cohort study; Level of evidence 3.

**Methods:**

Data were obtained from the Norwegian Knee Ligament Register. Patients undergoing primary ACL reconstruction (ACLR) between 2004 and 2024 were included for graft-survival analyses, and those operated by December 31, 2022, were eligible for 2-year Knee injury and Osteoarthritis Outcome Score (KOOS) outcomes. Patients with concomitant ligament injuries other than to the MCL, ACL repairs, or those with unknown ACL graft type were excluded. Patients were stratified into isolated ACL and ACL + MCL injury groups. Kaplan-Meier survival analyses (unadjusted), multivariable Cox regression, and linear/logistic regression models were used, adjusted for descriptive data, body mass index, meniscal/cartilage injury, time to surgery, smoking, pivoting sports, graft choice, and baseline KOOS.

**Results:**

A total of 35,139 ACLRs were included (2410 ACL + MCL and 32,729 isolated ACL; mean age, 28.6 years). Concomitant MCL injury was not associated with an increased overall risk of ACL revision (*P* = .5). However, ACL + MCL patients had lower 2-year KOOS Sport/Recreation (Sport/Rec) scores (−2 points, *P* = .04) and lower odds of achieving patient acceptable symptom state (PASS) for Sport/Rec. Within the ACL + MCL subgroup, hamstring (Hazard ratio [HR], 2.3; *P* = .001) and quadriceps autografts (HR, 3; *P* = .009) were associated with a higher revision risk compared with bone-patellar tendon-bone (BPTB) autograft, particularly when hamstring grafts were combined with nonoperative MCL treatment (HR, 2.4; *P* = .001). Graft choice did not influence the likelihood of achieving a minimal clinically important difference in KOOS.

**Conclusion:**

Concomitant MCL injury did not increase the risk of ACL revision but was associated with inferior KOOS Sport/Rec outcomes and lower odds of achieving PASS at 2 years. In patients with concomitant nonoperatively treated MCL injuries, BPTB autograft was associated with a significantly lower revision risk compared with hamstring and quadriceps autografts.

The medial collateral ligament complex (MCL) is the primary restraint to valgus laxity of the knee and an important secondary restraint to anterior translation and axial rotations.^4,5,27^ It is the most frequently injured ligament of the knee and is commonly associated with concomitant anterior cruciate ligament (ACL) injury.^5,7,19,38^ The treatment strategy for the MCL in such combined injuries is influenced by the clinical grade of instability and location of the MCL injury.^7^ While some authors recommend nonoperative treatment in most cases, the understanding of MCL anatomy, biomechanics, injury, and treatment strategies is changing. Thus, the optimal treatment for concomitant MCL injuries in the context of ACL rupture remains controversial.^7,17,37^ In the combined ACL/MCL setting, persistent or residual medial and anteromedial instability may place increased strain on the ACL graft, thereby increasing the risk of graft failure.^1,2,29^ Consequently, operative treatment of the MCL, either acute repair or reconstruction, is undertaken in selected cases to restore knee stability and the normal kinematic pattern of the knee.^9,20,30^ However, there is very little clinical evidence to guide the decision-making process, and practice guidelines report limited strength in their recommendation due to low-quality clinical evidence.^31^

In a systematic review, Jackson et al^13^ demonstrated an increased risk of subsequent ACL revision in nonoperatively treated concomitant MCL injuries. Ahn et al^1^ suggested up to a 13-fold increase in risk of ACL graft failure with unaddressed MCL laxity, and Alm et al^2^ a 17-fold increase.

A study from the Swedish National Knee Ligament Registry (SNKLR) supports these findings and, furthermore, MCL patients reported a statistically significantly lower Knee injury and Osteoarthritis Outcome Score (KOOS) at the 2-year follow-up.^32^ However, whether the difference in KOOS scores between patients with isolated ACL injury and those with ACL and concomitant MCL injury is clinically relevant remains unknown.

The present study aimed to evaluate patient-reported outcome measures (PROMs) and the risk of later ACL revision surgery in patients with concomitant MCL injuries at the time of ACL reconstruction (ACLR) compared with patients with isolated ACL injuries. Furthermore, the study aimed to evaluate whether the MCL treatment strategy (nonoperative vs operative) or ACL graft choice influenced PROMs or the risk of revision.

## Methods

This study was conducted in accordance with the RECORD (the Reporting of Studies Conducted Using Observational Routinely Collected Health Data) statement.^6^

### Patient Population

**Figure 1.**
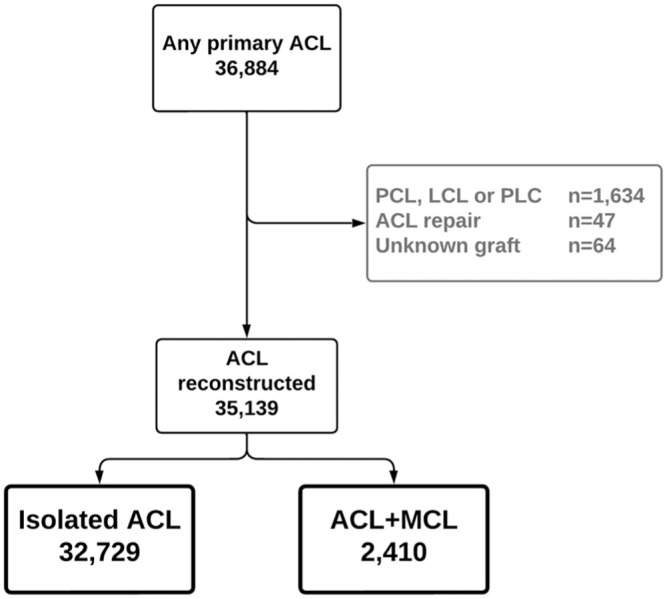
Flowchart illustrating the inclusion process. ACL, anterior cruciate ligament; LCL, lateral cruciate ligament; MCL, medial collateral ligament; PCL, posterior cruciate ligament; PLC, posterolateral ligament complex.

Patients included in the present study were identified from the Norwegian Knee Ligament Register (NKLR). The NKLR was established in 2004 and includes data on approximately 90% of cruciate ligament surgeries undertaken in Norway.^8,22^ Any subsequent ipsilateral knee surgery, such as revision, is reported to the NKLR by the treating surgeon. Patients registered with primary ACL surgery performed between 2004 and 31 December 2024 were eligible for inclusion in the graft survival analysis. In the analysis of the 2-year PROM and treatment failure, only patients who had undergone surgery before December 31, 2022, were eligible for inclusion to ensure adequate follow-up time. Patients with posterior cruciate ligament, concomitant lateral collateral ligament, or posterolateral ligament complex injuries were excluded. Furthermore, patients without ACLR (eg, ACL repair) or an unknown ACL graft status were excluded. The inclusion process is shown in Figure 1. Included patients were stratified into 2 groups: The *Isolated ACL* group (ACL as the only injured knee ligament) and the *ACL + MCL* group, consisting of patients who underwent ACL surgery and had a concomitant MCL injury, as reported by the surgeon. The MCL injury was treated surgically (repair and/or reconstruction) in some and nonoperatively in others. Nonoperative treatment of the MCL injury was defined as cases in which the treating surgeon reported an MCL injury. Still, no surgical intervention for the MCL was performed at the time of ACL surgery.

From the NKLR, the following variables were retrieved: sex; age; body height and weight; time of injury and ACL surgery; activity at the time of injury; any concomitant knee injuries, such as cartilage or meniscal injuries; KOOS—including the 5 subscales Pain, Knee-related Quality of life (QoL), Sports and Recreation, Activities of Daily Living (ADL), and Other Symptoms) preoperatively and at 2-year follow-up; smoking status; ACL graft choice; MCL treatment strategy; and any need for revision ACL surgery. The NKLR does not include any details on the localization or grading of MCL injuries or the rationale for choosing operative or nonoperative treatment.

### Statistics

The characteristics of the included patients at the time of ACL surgery in the 2 groups were compared using the Student *t* test or the chi-square test, as appropriate.

The risk of later ACL revision surgery was analyzed using the unadjusted Kaplan-Meier method and Cox regression models adjusted for sex, age at surgery, body mass index (BMI), concomitant meniscal injury, concomitant cartilage injury, time from injury to surgery, smoking status, and pivoting sports at injury according to Hefti et al.^10^

Multiple linear regression models were used to evaluate the differences in the KOOS subscales at the 2-year follow-up. Multiple logistic regression models were used to evaluate the odds of achieving improvement in KOOS Sport/Recreation (Sport/Rec) and Quality of Life (QoL) above the minimal clinically important difference (MCID) at the 2-year follow-up, and the odds of treatment failure during a 2-year follow-up. The KOOS subscale MCID thresholds suggested by Ingelsrud et al^12^ were used. Treatment failure was defined as (1) having undergone revision surgery or (2) failing to achieve the patient acceptable symptom state (PASS) at the 2-year follow-up for the KOOS Sports/Rec subscale. The KOOS subscale PASS thresholds suggested by Urhausen et al^36^ were used. The KOOS Sport/Rec and QoL subscales were used because previous studies have demonstrated that they are the most responsive and discriminative among patients with ACL injury.^12,32,35^

The regression models were adjusted for sex, age at surgery, BMI, concomitant meniscal injury, concomitant cartilage injury, ACL graft, time from injury to surgery, smoking status, pivoting sports at the time of injury, and the respective preoperative KOOS subscale.

Patients with missing data for the adjusted variables were listwise deleted from the regression models. Statistical significance was set a priori at *P* < .05. STATA 18 (StataCorp LLC) was used for the statistical analysis.

### Ethics

The NKLR has been approved by the Norwegian Data Protection Authority to collect, analyze, and publish health data on patients with knee ligament injuries, provided that patients have given informed consent. No further ethical approval was necessary, according to an earlier evaluation by the regional ethics committee.^8^

## Results

The NKLR database contained 36,884 eligible primary ACLRs, of whom 35,139 met the inclusion criteria (Figure 1). There were 2410 injuries involving combined ACL and MCL injury, and 32,729 injuries in which an ACL tear was the only ligament injury. The mean age at the time of ACL surgery was 28.6 years (95% CI, 25.5-28.7), and the mean time from injury to ACL surgery was 1.6 years (95% CI, 1.5-1.6). Only 18.5% of the patients underwent ACL surgery within 3 months of their knee injury. This indicates that most ACLRs in the present cohort were performed in a subacute or chronic setting rather than acutely after injury. There were statistically significant differences in the characteristics of the 2 study groups at the time of surgery (Table 1). Furthermore, the preoperative KOOS subscales were significantly lower in the ACL + MCL group than in the isolated ACL group (Table 1).

**Table 1:** Characteristics at the Time of Index ACL Surgery*^*a*^*

	N = 35,139	Isolated ACL, %	ACL + MCL, %	*P*
Sex M/F	19,169/15,970	54.6/45.4	53.8/46.1	.5479
Mean age, years		28.4	31.1	<.001
BMI, kg/m^2^	27,435	25	25.4	<.001
Meniscal injury	20,263	57.7	56.1	.001
Cartilage injury	8279	23	30.4	<.001
Time injury-surgery, years		1.6	0.9	<.001
Follow-up, years		8.9	8.3	<.001
Pivoting sports	26,195	77.9	70	<.001
Smoking				
No	23,019	84.8	83.4	
Occasional	2489	9.1	9.3	.195
Daily	1657	6	7.1	
ACL graft				<.001
BPTB	17,550	15,937 (48.7)	1613 (66.9)	
Hamstring	16,197	15,532 (47.5)	665 (27.6)	
Allograft	47	30 (0.1)	17 (0.7)	
Quadriceps	1340	1225 (3.7)	115 (4.8)	
KOOS preop				
Symptoms	26,464	71.5	70.6	.046
Pain	26,227	73.4	72	.002
ADL	26,169	81.9	79.3	.000
Sport/Rec	26,106	43.3	38.8	.000
QoL	24,461	35.1	33.7	.002
MCL treatment				
Nonop			1969 (81.7)	
Repair			107 (4.4)	
Reconstruction			334 (13.9)	

a Data are presented as n (%), unless otherwise indicated. ACL, anterior cruciate ligament; ADL, activities of daily living; BMI, body mass index; BPTB, bone-patellar tendon-bone; KOOS, Knee injury and Osteoarthritis Outcome Score; MCL, medial collateral ligament; M/F, male/female; Postop, postoperative; Preop, preoperative; QoL, quality of life; Rec, recreation.

### Influence of Concomitant MCL Injury on Risk of Revision and PROM at 2 Years

**Figure 2.**
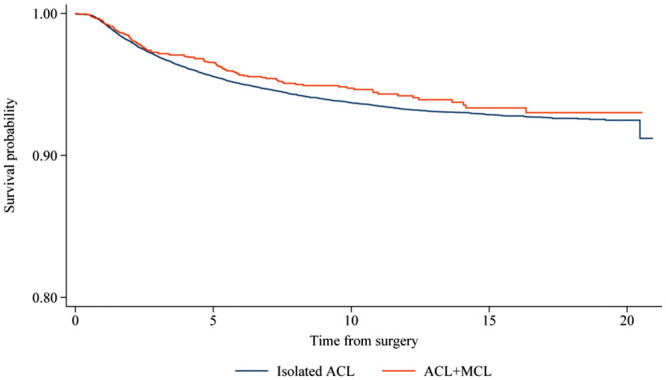
Survival curves illustrating the risk of later ACL revision surgery in the 2 study groups. ACL, anterior cruciate ligament.

**Figure 3.**
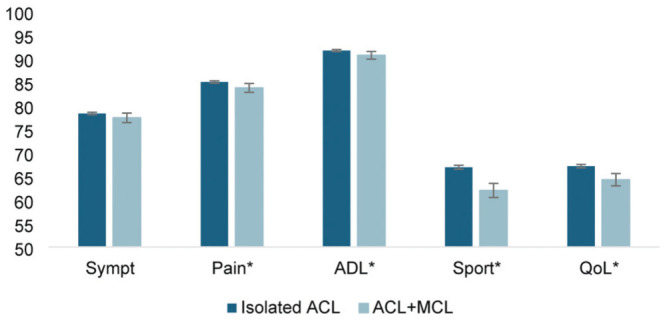
KOOS scores for both *isolated ACL* and *combined injury* groups at 2-year follow-up. Whiskers represent 95% CIs. The asterisks indicate a statistically significant difference. ADL, activities of daily living; KOOS, Knee injury and Osteoarthritis Outcome Score; QoL, quality of life; Rec, Recreation.

Key adjusted results for revision risk, MCID, and PASS are summarized in Table 2, while differences in KOOS subscales are illustrated in Figures 2 and 3. The presence of a concomitant MCL injury did not influence the risk of later ACL revision surgery overall, as illustrated by the Kaplan-Meier curve in Figure 2 and the Cox regression model (*P* = .5) in Table 2. The odds of achieving PASS at the 2-year follow-up were, however, significantly lower for the KOOS sport/rec subscale in the ACL + MCL group (Table 2). Furthermore, in an adjusted regression model, ACL + MCL patients had a mean KOOS Sport/Rec score 2 points lower (95% CI, –3.8 to −0.1; *P* = .04) at the 2-year follow-up. No significant differences were observed in the other KOOS subscales. Due to ceiling effects in the KOOS subscales, a Tobit regression model was used to verify these findings. The odds of achieving PASS for QoL and of achieving improvement above MCID for the Sport/Rec and QoL subscales were not influenced by a concomitant MCL injury (Table 2). The KOOS subscales at the 2-year follow-up are presented in Figure 3. There were significant but small differences across all subscales, except for KOOS symptoms.

**Table 2:** Risk of Subsequent ACL Revision Surgery and Odds of Achieving MCID and PASS at 2-Year Follow-up*^*a*^*

		Crude			Adjusted*^*b*^*		
	N	Isolated ACL	ACL + MCL	*P*	Isolated ACL	ACL + MCL	*P*
Risk of revision, HR*^*c*^*	1878	Ref	0.9 (0.7-1.1)	.173	Ref	1.1 (0.9-1.4)	.471
KOOS MCID, OR*^*d*^*							
Sport/Rec	9154	Ref	1.1 (0.9-1.2)	.366	Ref	0.9 (0.8-1.1)	.479
QoL	10733	Ref	1.1 (0.9-1.3)	.281	Ref	1.1 (0.9-1.3)	.252
KOOS PASS, OR*^*d*^*							
Sport/Rec	9056	Ref	0.7 (0.6-0.8)	**<.001**	Ref	0.7 (0.6-0.8)	**<.001**
QoL	12317	Ref	0.9 (0.8-1)	.008	Ref	1 (0.8-1.1)	.724

a Data are presented as mean (95% CI). Boldface *P* values indicate statistical significance. ACL, anterior cruciate ligament; BMI, body mass index; HR, hazard ratio from Cox model; MCL, medial collateral ligament; MCID, minimal clinically important difference; OR, odds ratio from logistic regression model; PASS, patient acceptable symptom state; KOOS, Knee injury and osteoarthritis outcome score; QoL, quality of life; Rec, recreation; Ref, reference.

b Adjusted for sex, age at surgery, BMI, concomitant meniscal injury, concomitant cartilage injury, time from injury to surgery, smoking status, and pivoting sports at injury.

c Cox regression model.

d Logistic regression model, adjusted for the respective preoperative KOOS score, in addition to*^b^*.

### Subgroup Analysis of Patients With Concomitant MCL Injury

**Figure 4.**
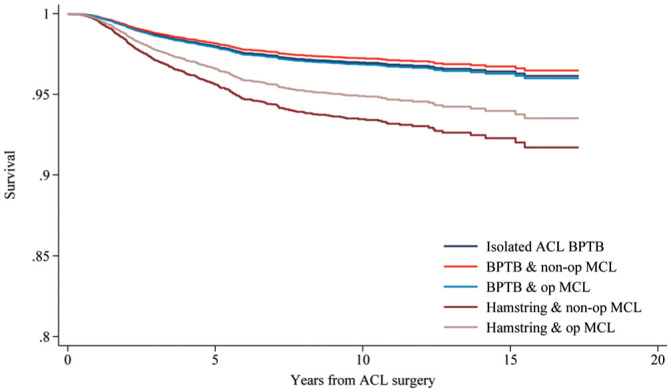
Adjusted survival curves illustrating the risk of subsequent ACL revision surgery based on ACL graft choice and MCL treatment strategy with isolated ACLR as reference. Survival analysis adjusted for sex, age at surgery, BMI, concomitant meniscal injury, concomitant cartilage injury, time from injury to surgery, smoking status, and pivoting sports at injury. ACL, anterior cruciate ligament; ACLR, ACL reconstruction; BMI, body mass index; BPTB, bone-patellar tendon-bone; MCL, medial collateral ligament; Nonop, nonoperative.

ACLR using hamstring or quadriceps tendon autografts in the presence of a concomitant MCL injury was associated with a significantly higher risk of subsequent ACL revision than when using bone-patellar tendon-bone (BPTB) autograft (Table 3). Patients who underwent ACLR with a hamstring graft in combination with a nonoperatively treated MCL injury had a significantly higher risk of subsequent ACL revision than those who underwent ACLR with BPTB and nonoperative MCL treatment (Table 4 and Figure 4). However, surgical treatment of concomitant MCL injuries did not influence the risk of revision in either hamstring or BPTB ACLR (Table 4). Furthermore, there were no significant differences in the odds of achieving an improvement above the MCID at the 2-year follow-up in KOOS Sport/Rec or QoL among the four treatment groups. In addition, no significant differences were found in the odds of treatment failure at 2 years (Table 4).

**Table 3:** Risk of Revision of ACL Based on Graft Choice in Patients With Concomitant MCL Injury*^*a*^*

	N	Crude		Adjusted*^*b*^*	
	Graft/Revisions	HR	*P*	HR	*P*
ACL graft choice					
BPTB	1613/51	1		1	
Hamstring	665/55	2.1 (1.5-3.1)	**<.001**	2.3 (1.4-3.8)	**.001**
Quadriceps	115/8	2.9 (1.4-6.2)	**.005**	3 (1.3-7)	**.009**
Allograft	17/0	00 (0-NA)	≥.999	00 (0-NA)	≥.999

a Data are presented as mean (95% CI). Boldface *P* values indicate statistical analysis. ACL, anterior cruciate ligament; BMI, body mass index; BPTB, bone-patellar tendon-bone; MCL, medial collateral ligament; NA, not applicable.

b Adjusted for sex, age at surgery, BMI, concomitant meniscal injury, concomitant cartilage injury, time from injury to surgery, smoking status, and pivoting sports at injury.

**Table 4:** Risk of Subsequent ACL Revision or Treatment Failure at 2 Years and Odds of Achieving MCID at 2 Years Based on ACL Graft Choice and MCL Treatment Strategy*^*a*^*

				MCID at 2 Years*^*d*^*						
	Revision*^*b*^*			Sport/Rec		QoL		Failure*^*d*,*e*^*		
	%*^*c*^*	Adjusted HR	*P*	Adjusted OR	*P*	Adjusted OR	*P*	%*^*c*^*	Adjusted OR	*P*
BPTB + nonop MCL	3.7	Ref		ref		Ref		58.8	Ref	
BPTB + MCL	1.7	1.1 (0.5-2.7)	.830	0.9 (0.5-1.6)	0.675	0.8 (0.4-1.6)	.548	76.2	1.6 (0.9-2.8)	.077
Hamstring + nonop MCL	9.6	2.4 (1.5-3.9)	**.001**	0.9 (0.6-1.4)	0.756	0.9 (0.6-1.4)	.573	59.7	1.1 (0.8-1.6)	.630
Hamstring + MCL	2.2	1.9 (0.3-13.7)	.546	0.7 (0.2-2.2)	0.502	0.5 (0.1-1.8)	.256	60.7	0.9 (0.3-3)	.909

a Data are presented as mean (95% CI). The boldface *P* value indicates statistical significance. ACL, anterior cruciate ligament; BMI, body mass index; BPTB, bone-patellar tendon-bone graft; HR, hazard ratio; KOOS, Knee injury and Osteoarthritis Outcome Score; MCL, medial collateral ligament; nonop, nonoperative; OR, odds ratio; Rec, recreation.

b HR from a Cox regression model adjusted for sex, age at surgery, BMI, concomitant meniscal injury, concomitant cartilage injury, time from injury to surgery, smoking status, and pivoting sports at injury.

c Percentage of patients in each group meeting the outcome.

d Adjusted OR from logistic regression adjusted for the respective preoperative KOOS subscale as well as the variables from *^b^*.

e Failure defined as failure to achieve a patient-acceptable symptom state for the KOOS Sport/Rec subscale or having undergone ACL revision within 2 years.

## Discussion

The main findings of this study of a large nationwide cohort were that concomitant MCL injury in the setting of ACLR did not influence the risk of subsequent ACL revision or the odds of achieving MCID overall. However, ACLR with a hamstring graft in patients with concomitant MCL injury significantly increased the risk of subsequent ACL revision, especially when combined with nonoperative treatment of the MCL injury. Furthermore, concomitant MCL injury significantly lowered the odds of achieving PASS in the KOOS Sport/Rec subscale. However, the treatment strategy for concomitant MCL injury did not influence the odds of achieving a KOOS improvement above MCID or of avoiding treatment failure at the 2-year follow-up.

In their registry and large database studies, Svantesson et al^32^ and Niknam et al,^21^ respectively, reported a 32% to 50% increased risk of ACL revision in patients with concomitant MCL injury compared with patients with isolated ACL.^3^ This contrasts with the finding of no difference in the odds of revision between the groups in the present study. The NKLR and the studies by Svantesson et al and Niknam et al lack information on the localization and grading of concomitant MCL injuries. This limitation may result in substantial between-study differences in MCL treatment protocols, as both injury localization and severity influence treatment strategy.^7^ Neither Niknam et al nor Svantesson et al reported any details on the graft used for ACLR. Still, differences in ACL graft choice could partly explain the higher revision risk observed in their studies compared with the present study, especially if their patients predominantly received hamstring ACL grafts. Both Samuelsen et al^28^ and Zhao et al^40^ demonstrated a higher risk of ACL revision with hamstring autograft ACLR, and, at least in the Swedish NKLR, the proportion of hamstring grafts is substantially higher (94%) than in our NKLR cohort (46%).^26^ Several studies have suggested that the hamstring tendons play an important role as secondary valgus stabilizers in MCL-deficient knees and that valgus instability increases the strain on the ACL.^11,14,18^ This is consistent with the increased risk of subsequent ACL revision in patients with combined ACL and MCL injuries treated with hamstring ACLR, as found in the present study. Quadriceps tendon autografts were also associated with an increased risk of subsequent ACL revision in patients with concomitant MCL injury as compared with patellar tendon ACL grafts. The increased risk of revision in the recently popularized quadriceps grafts is well known from a previous study from the NKLR.^39^ It could partly be due to the learning curve of the technique. Furthermore, most quadriceps patients were operated on at 2 hospitals, with a higher proportion of high-level athletes, who have higher rerupture rates than the general population.^15,23,39^

MCID and PASS are considered more robust methods for evaluating the clinical relevance of differences in PROM between groups, but require that validated MCID and PASS thresholds have been established in the same population.^16^ Therefore, the MCID and PASS thresholds from ACL cohorts in Norwegian patients with ACL injury were used.^12,36^ To the best of our knowledge, this is the first study to report the proportion of patients with ACL injury and concomitant MCL injury who achieved the MCID and PASS. The differences in KOOS subscales between the isolated ACL and ACL/MCL patients (Figure 3) were statistically significant but small. This supports our finding of no significant difference in the odds of achieving improvement above the MCID between the 2 groups. However, ACL/MCL injury was associated with lower odds of achieving PASS on the KOOS Sport/Rec subscale, underscoring the implications of this injury pattern, which is associated with greater severity than isolated ACL rupture. This finding is consistent with the report by Svantesson et al, who found that only 10% of patients with ACL/MCL injury returned to their preinjury level of sport 1 year postoperatively.^34^

In their SNKLR study, Svantesson et al^32^ demonstrated that nonoperative treatment of a concomitant MCL injury at the time of ACLR significantly increased the risk of subsequent ACL revision surgery. In the present study, only the combination of hamstring ACLR and nonoperative MCL treatment was associated with a significantly increased risk of revision. This suggests that using ipsilateral hamstring tendons without addressing concomitant MCL insufficiency may not be an appropriate treatment strategy. Nevertheless, this contrasts with findings from another SNKLR study showing no difference in revision risk among patients with ACL + MCL injuries in whom the MCL component was managed nonoperatively, when comparing hamstring and BPTB ACL grafts.^33^ However, the increased risk of revisions in hamstring ACLRs is well known, and the latter SNKLR study is hampered by a low number of patients having BPTB grafts, making it prone to underestimating the difference.^28,33,40^ In the analysis of the risk of treatment failure, defined as subsequent revision or failure to achieve KOOS Sport/Rec PASS within 2 years, no significant differences were found between the treatment groups. This suggests lower odds of achieving PASS in the KOOS Sport/Rec subscale in the BPTB group, as also reported by Svantesson,^33^ including both revision and failure to achieve PASS in the failure analysis, may increase the validity of the findings of the present study, as not all patients with an inadequate ACL graft undergo revision surgery.

This study had several limitations. The most important limitation is that the included patients were not randomly allocated to the treatment groups. Although the statistical analyses were adjusted for known significant baseline differences, other significant confounders may have remained, biasing the results. The NKLR does not contain any information on the localization or grading of concomitant MCL injury, and there could be significant differences in the severity of the concomitant MCL injuries. Furthermore, the NKLR does not provide the rationale for the chosen treatment strategy or details of the nonoperative treatment for the MCL component of the injury. Additionally, the majority of ACLRs in the present cohort were performed in a subacute or chronic setting rather than acutely after injury. This distinction should be considered when interpreting the results. KOOS was available in only 74% of patients at the 2-year follow-up; thus, attribution bias may be present. Previous studies have demonstrated that the minor differences in PROM scores between ACL patients who responded at follow-up and those who did not have limited clinical relevance.^24,25^ A register study, such as the present one, should be considered hypothesis-generating, and the findings may not be representative of other populations. Large, nonoperative, multicenter randomized controlled trials are needed to identify the optimal treatment for combined ACL and MCL injuries.

## Conclusion

In a large cohort of patients from the general population undergoing ACLR, a concomitant MCL injury was not associated with an increased risk of subsequent ACL revision overall but was associated with lower odds of achieving PASS at 2 years. However, in patients with concomitant nonoperatively treated MCL injuries, ACLR with hamstring and quadriceps autografts was associated with a higher risk of revision compared with BPTB autografts.
